# *Paraphysoderma sedebokerense* GlnS III Is Essential for the Infection of Its Host *Haematococcus lacustris*

**DOI:** 10.3390/jof8060561

**Published:** 2022-05-25

**Authors:** David Alors, Kevin R. Amses, Timothy Y. James, Sammy Boussiba, Aliza Zarka

**Affiliations:** 1 Microalgal Biotechnology Laboratory, French Associates Institute for Agriculture and Biotechnology of Drylands, The Jacob Blaustein Institutes for Desert Research, Sede-Boker Campus Ben Gurion University of the Negev, Beersheba 8499000, Israel; sammy@bgu.ac.il; 2 Departamento de Biología y Químicas, Facultad de Recursos Naturales, Campus San Juan Pablo II, Universidad Católica de Temuco, Temuco 478 0694, Chile; 3 Department of Ecology and Evolutionary Biology, University of Michigan, Ann Arbor, MI 48109, USA; amsesk@oregonstate.edu (K.R.A.); tyjames@umich.edu (T.Y.J.)

**Keywords:** *Paraphysoderma sedebokerense*, *Haematococcus lacustris*, glutamine synthetase, nitrogen metabolism, fungi, glufosinate

## Abstract

Glutamine synthetase (GlnS) is a key enzyme in nitrogen metabolism. We investigated the effect of the GlnS inhibitor glufosinate on the infection of *H. lacustris* by the blastocladialean fungus *P. sedebokerense*, assuming that interfering with the host nitrogen metabolism will affect the success of the parasite. Complete inhibition of infection, which could be bypassed by the GlnS product glutamine, was observed at millimolar concentrations of glufosinate. However, this effect of glufosinate was attributed to its direct interaction with the blastoclad and not the host, which results in development and growth inhibition of the blastoclad. In our *P. sedebokerense* draft genome, we found that the sequence of GlnS is related to another fungal GlnS, type III, found in many poor known phyla of fungi, including Blastocladiomycota and Chytridiomycota, and absent in the main subkingdom of fungi, the Dikarya. We further tested the ability of the blastoclad to utilize nitrate and ammonia as inorganic nitrogen sources and glutamine for growth. We found that *P. sedebokerense* equally use ammonia and glutamine and use also nitrate, but with less efficiency. Altogether, our results show that GlnS type III is mandatory for the development and growth of *P. sedebokerense* and could be an efficient target to develop strategies for the control of the fungal parasite of *H. lacustris*.

## 1. Introduction

The blastocladialean fungus *Paraphysoderma sedebokerense* is a facultative parasite that can also grow as a saprobe [1]. This fungus is economically important because of its ability to infect and crash cultures of the green microalga *Haematococcus lacustris,* known by its previously accepted synonym, *H. pluvialis* [2]. This alga is grown in large scale for the production of the ketocarotenoid astaxanthin, known as the best natural antioxidant with accepted therapeutic properties [3,4]. In addition, *P. sedebokerense* also infects *Chromochloris zofingiensis* and *Scenedesmus dimorphus* [5,6], which have economic potential as a source of pigments and lipids for use as nutritional complements or biofuel, respectively [7,8]. The microalga *H. lacustris* is, however, the preferred host for *P. sedebokerense* and suffers completely virulent, lethal infections under all tested conditions [5,6]. The economic impact of this epidemic is reflected in efforts to control the development of the *P. sedebokerense* infections by different strategies, such as selection of less sensitive strains [9,10], changing culture media [11], or using surfactants [12].

Current understanding of *P. sedebokerense* is limited to about a dozen research articles published since the identification of the species in 2008 [1]. The works are mainly focused on the taxonomy and life cycle of the blastoclad [1,13,14], its host range specificity, preference to *H. lacustris* [5,6,15], and recently, dual transcriptomic research describing the carbon metabolism of the blastoclad [16]. Recent findings showed the utilization of inorganic nitrogen sources [17], contradicting a previous assertion that only organic nitrogen can be utilized by *P. sedebokerense* [1]. This finding is particularly relevant to understanding the dynamics of fungal parasitism of algae since nitrogen is essential for fungal virulence [18], and nitrogen was shown to facilitate infection development in *H. lacustris* [19].

Nitrogen is an essential element because it is a component of both nucleic acids and proteins. Nitrogen availability greatly impacts plant–fungal systems [20], most importantly affecting plant growth and plant response to pathogens [21] and generally promoting fungal infection [22], but some fungal pathogens are partially or absolutely inhibited by excess of nitrogen [23], or differentially inhibited depending on nitrogen species [24]. The utilization of different nitrogen sources could influence morphological transitions and virulence factors in fungi [24,25]. The ability of zoosporic true fungi to utilize different nitrogen compounds is variable, and different from species to species; however, fourteen out of seventeen tested Chytridiomycota isolates grew on nitrate as the sole nitrogen source, but not the two tested Blastocladiomycota isolates, *Allomyces arbuscula* and *Catenaria anguillulae* [26].

Eukaryotes can assimilate nitrogen from various sources, but any inorganic nitrogen needs to be reduced to ammonia before it is assimilated into glutamate to form glutamine, by the enzyme glutamine synthetase (GlnS) in plants [27] and fungi [28]. Because of their importance, nitrogen assimilation routes are a classical target for the design of herbicides. One example of these herbicides is BASTA (AgrEvo, Frankfurt am Main, Germany), which contains the GlnS inhibitor glufosinate (also called phosphinothricin) as the active ingredient, which binds irreversibly to the catalytic domain of the GlnS. Inhibition of GlnS activity blocks the conversion of inorganic nitrogen to organic nitrogen and causes the death of the plant, due to the accumulation of ammonia inside the cell. When glufosinate was applied to cultures of the microalgae *H. lacustris,* it showed resistance to glufosinate, escaping the harmful effects by excreting ammonia, though glufosinate induced the accumulation of astaxanthin, catalyzing the transformation of cells from green vegetative cells to red resting cysts, as occurs during nitrogen starvation [29]. Two different decameric isoforms of glutamine synthetase were identified in *H. lacustris* i.e., a cytoplasmic GlnS2 holoenzyme termed GlnS2c, and a plastid form, termed GlnS2p [30].

The current study investigates the effect of glufosinate on the infection of *H. lacustris* by *P. sedebokerense*. We hypothesized that interfering with the host nitrogen metabolism pathway using glufosinate could help to control the infection and could further help to develop strategies to control the pest. This hypothesis was based on a previous report, indicating the inability of *P. sedebokerense* to utilize inorganic nitrogen [1].

## 2. Materials and Methods

### 2.1. Strains and Growth Conditions

We used the blastoclad *P. sedebokerense* isolate AZ_ISR (2019) isolated from Sede-Boker (Midreshet Ben Gurion, Israel) and considered it as a new isolate of the holotype strain MycoBank MB 561751 [1,13]. Pure cultures of blastoclad were grown in BGM in the presence of antibiotics at 30 °C in an incubator shaker (180 rpm) supplemented with 2% CO_2_, under continuous dim white light (15 mol photons m^−2^ s^−1^) illumination [19]. *H. lacustris* Flotow 1844 em. Wille K-0084 was obtained from the Norwegian Culture Collection of Algae, Oslo. *H. lacustris* axenic cultures, 2 × 10^5^ cells mL^−1^ (100 mL culture in 250 mL Erlenmeyer flasks), were grown for 7 days in modified BG-11 (mBG_11_) containing NaNO_3_ as the sole nitrogen source [31], at a controlled temperature (25 °C), with constant illumination (80 mol photon m^−2^·s^−1^), and in an incubator shaker (150 rpm) enriched with 300 mL·mim^−1^ CO_2_. For maintenance, *H. lacustris* cultures were diluted 10-fold weekly in fresh media to approximately 2 × 10^5^ cells mL^−1^. At these cell densities, cultures reach stationary stage (2 × 10^6^ cells/mL) after one week. This “green culture” was routinely used. The “red culture” was obtained by replacing NaNO_3_ in the medium with an equivalent molar concentration of NaCl.

### 2.2. Infection Conditions and Effect of Glufosinate on P. sedebokerense Growth and Infection Development

Infection tests were conducted with pure *P. sedebokerense* propagules. Fresh harvested propagules were obtained from stationary blastoclad cultures (7–10 days old) by filtration, following the method previously developed in our laboratory [19]. All infection tests were carried out in CELLSTAR^®^ suspension 6-well plates (Greiner Bio-One, Frickenhausen, Germany) by inoculating 5 × 10^5^ propagules/well (3 mL culture in each well) with the same number of 7-day-old *H. lacustris* cells (stationary stage) in mBG_11_ culture media. Co-cultures were incubated at 30 °C in an incubator shaker (180 rpm) supplemented with 2% CO_2_, under continuous dim white light (15 mol photons m^−2^ s^−1^) illumination. The inoculated cultures were checked daily and maintained for a minimum of seven days.

The herbicide glufosinate-ammonium was used to inhibit the *P. sedebokerense* growth and infection development in *P. sedebokerense–H. lacustris* co-cultures. To test the effect on growth and infection, the herbicide was added to final concentrations of 0.1, 0.5, 1, and 2.5 mM. In the control culture, NH_4_Cl (2.5 mM) was added, and pH was measured at the end of the experiments, verifying that it was not different between treatments.

We also designed one assay to test if the effect of glufosinate on *P. sedebokerense* infected *H. lacustris* culture was due to the irreversible inhibition of the algal GlnS enzyme or the fungal GlnS, by pretreating green and red *H. lacustris* cells with glufosinate. To this end, 10 mL culture of *H. lacustris* was inoculated in the presence of 2.5 mM glufosinate 7 days, under the above described algal growth conditions. We then pelleted the cells (2200 g, 5 min), discarded the supernatant, and washed the cells two times before adding fresh culture medium to eliminate residues of glufosinate in the infected culture; these cells were used for infection by *P. sedebokerense* in the absence of glufosinate.

### 2.3. Testing the Effect of Nitrate, Ammonia, and Glutamine as Nitrogen Sources on Blastoclad Growth

To test the growth with different nitrogen compounds we used BGM w/o nitrogen. This medium was formulated as follows: we prepared a modified BGM medium without peptone and replaced the yeast extract with yeast extract without amino acids, ammonium sulphate, and para-amino benzoic acid (Formedium, Hunstanton, Norfolk, UK); this yeast extract contained vitamins, trace elements, and salts. To keep the pH of the culture media at 7.5 as in BGM, we replaced HEPES buffer with the same concentration (20 mM) of MOPS buffer. The assayed nitrogen compounds were nitrate (NaNO_3_), ammonia (ClNH_4_), and glutamine. To avoid bacterial contamination, we used kanamycin (0.15 mg L^−1^) and ampicillin (0.15 mg L^−1^). Nitrogen compounds were added to three different final concentrations of 5, 15, and 25 mM nitrogen equivalents to compare with the results of Hoffman et al. [1].

The assays were done in nitrogen free CELLSTAR^®^ 12-well suspension plates (Greiner Bio-One, Frickenhausen, Germany) by triplicate, by inoculating up to 1 mg of the wet weight of propagules (2 × 10^5^ cells) in two milliliters of culture media. The growth was qualitatively assessed by taking pictures of the 12-well plates and quantitatively assessed by measuring the wet weight in mg. Statistical differences between treatments was compared by confidence intervals at α = 0.5 with the formula CI 95 = (average-*t*) × (desvest./SQRT (*n*), where *t* is the value of student t for α/2 and *n* the sample size.

### 2.4. Glutamine Synthetase Activity

Aliquots of 10 mL 3 days old *P. sedebokerense* cultures or pure propagules (40 mg wet weight) were harvested by centrifugation (14,000× *g* for 5 min), resuspended in 0.5–1 mL of 20 mM tris [hydroxymethyl]-aminomethane hydrochloride pH 7.0 buffer, and homogenized. The resulting homogenate was used as a source of the GlnS enzyme. The activity was determined by the transferase assay, where the formation of y-glutamyl hydroxamate is measured [32]. Since we did not detect activity with this assay, we used the direct biosynthetic GlnS assay, where the liberated phosphate was determined via the formation of phosphomolybdate complex [33]. Protein was determined after Bradford [34], using bovine serum albumin as a standard.

### 2.5. Genome Sequencing, GlnS Predicted Protein Searching and Protein Phylogenetic Analysis

Total DNA of the AZ_ISR strain of *P. sedebokernse* was extracted from 40 mg (wet weight) of isolated propagules [19] using the Plant Genomic DNA Mini Kit (Geneaid, New Taipei City, Taiwan). Short-read libraries were prepared using NEBNext Ultra reagents and sequenced on 5% of an Illumina NovaSeq (S4), generating 300 cycles of 150 bp paired- end reads. This portion of the flow cell yielded 42,763,435 paired-end reads, which, after trimming and quality filtering with cutadapt [35], were assembled with dipSPAdes [36]. After removing contigs shorter than 500 bp, the *P. sedebokernse* AZ_ISR assembly consisted of 25.43 Mbp on 427 contigs (N50 = 164,841 bp) with a GC content of 41.24% and CEGMA-estimated [37] completeness of 92.74%. A total of 7463 proteins were predicted on contigs with funannotate v1.7.2 [38] with the predicted proteome of *P. sedebokernse* JEL821 supplied as supporting evidence for annotations [39]. After identifying GlnS III in *P. sedebokerense*, we used BLAST to recover related fungal sequences from NCBI. Outgroup taxa were chosen based on [40]. Proteins were aligned using a MAFFT v. 7 online server using the G-INS-I option. Ambiguously aligned regions were removed using Gblocks 0.91b [41], with default options except retaining aligned residues with 50% or fewer gapped sequences. The phylogeny was estimated using RAxML version 8.2.11 [42], with options -f a, -m PROTGAMMAILG, and 1000 bootstrap pseudo-replicates for clade support.

## 3. Results

### 3.1. Effect of Glufosinate on H. lacustris Infection by P. sedebokerense

**Figure 1:**
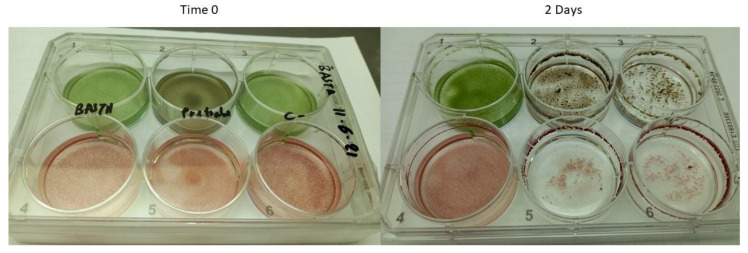
Effect of 2.5 mM of glufosinate on the infection of *H. lacustris* by *P. sedebokerense*. Epidemic development in green (upper row) and red nitrogen starved (lower row) *H. lacustris* culture, two days after inoculation with 5 × 10^5^ *P. sedebokerense* propagules, at a propagules/host ratio of 1:1. We tested the epidemic development in the presence of 2.5 mM glufosinate (left column of each plate), in *H. lacustris* cells pretreated with 2.5 mM glufosinate (central column of each plate) and in the absence of glufosinate (negative control) (right column of each plate). Left plate, zero-time control; right plate, same plate after two days (lid was removed before imaging).

*H. lacustris* green cells (at the stationary stage) or red aplanospores (induced by nitrogen starvation) inoculated with pure *P. sedebokerense* propagules at a ratio of 1:1 (cell/cell) fully collapsed 48 h after inoculation (Figure 1, right column of the plates). However, when 0.1–2.5 mM glufosinate was added to the co-cultures at the onset of cultivation, a dose-dependent inhibitory effect of the infection was observed. Complete inhibition of the infection was observed with 2.5 mM glufosinate (Figure 1, left column of the plates), and this treated culture was not different from a control culture in the absence of the blastoclad. Lower concentrations of glufosinate, i.e., 0.1, 0.5, and 1.25 mM showed partial inhibition of the infection (Figure S1). Therefore, we conducted the infection assays at a glufosinate concentration of 2.5 mM. Similar glufosinate concentration dependence was previously described for the inhibition of *H. lacustris* GlnS activity [29].

Based on the previous report of Hoffman et al. [1], indicating the inability of *P. sedebokerense* to utilize inorganic nitrogen compounds, we hypothesized the observed inhibitory effect of glufosinate on the infection is due to its direct effect on *H. lacustris,* indirectly affecting the success of the fungus (by interrupting its nitrogen availability), instead of directly affecting fungal growth. To test this hypothesis, we incubated *H. lacustris* with 2.5 mM glufosinate for a period of one week, washed the cells, and replaced the medium with glufosinate-free fresh algal growth medium before inoculation with *P. sedebokerense*. We refer to these algae as “pre-treated cells”, since glufosinate is known to act as an irreversible inhibitor. Our results (Figure 1, middle column, left plate) show that upon pretreatment with glufosinate, green *H. lacustris* cultures turned brown, due the accumulation of the red pigment astaxanthin, as previously reported [29]. This change of color together with arrested growth relative to the control indicate the successful inhibition of GlnS activity in the pre-treated cells. Two days after inoculation with the blastoclad, both green and red pre-treated cells developed epidemics and the culture collapsed. (Figure 1 middle column, right plate). These results led us to conclude that the inhibitory effect of glufosinate on the infection of *P. sedebokerense* is due to its effect on the blastoclad’s GlnS and not on the host GlnS; glufosinate needs to be present in the co-culture to exert its effect on the infection.

**Figure 2:**
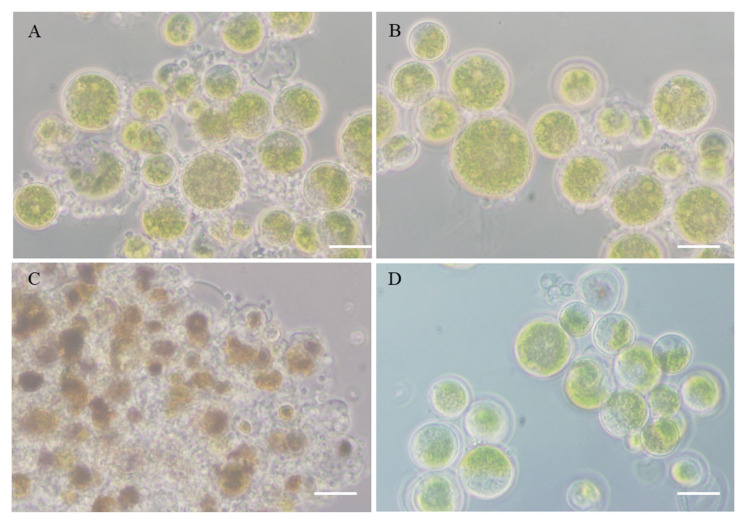
Microscopic images of *P. sedebokerense* infection development in *H. lacustris* cultures, in the absence (**A**,**C**) or presence (**B**,**D**) of 2.5 mM glufosinate. (**A**,**B**), and (**C**,**D**) The upper images were taken 3 h after inoculation, whereas the bottom images were taken 9 days after inoculation of the infecting the co-cultures. Glufosinate did not prevent the encystment of the propagules 3 h after inoculation (**B**) but prevented the development of the infection and the collapse of the algal culture up until 9 days (**D**). Scale bar 20 μm.

At the microscopic level, glufosinate reduced but did not completely prevent the encystment of *P. sedebokerense* propagules on *H. lacustris* cells, as was observed a few hours after the inoculation (Figure 2A,B). The efficiency of glufosinate in disease prevention is clear from the comparison of the control, featuring a totally collapsed algal culture (Figure 2C) compared with the culture treated with glufosinate remaining green with most of the algal cells uninfected and healthy nine days after inoculation (Figure 2D). In this case, the cells did not accumulate significant amounts of astaxanthin, respectively, and did not change their color since the light intensity during the exposure to glufosinate was very low.

### 3.2. Glufosinate Targets the GlnS Enzyme to Inhibit H. lacustris Infection by P. sedebokerense

**Figure 3:**
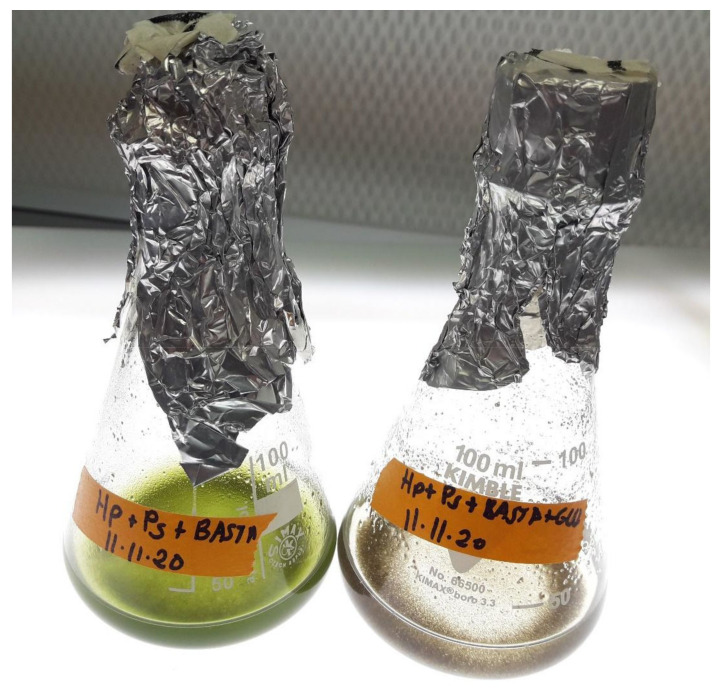
Addition of glutamine abolishes the inhibitory effect of glufosinate on the infection of *H. lacustris* by *P. sedebokerense* propagules. Shown are flasks of *H. lacustris* and *P. sedebokerense* cocultures after 7 days. Left: coculture in the presence of 2.5 mM of glufosinate; Right: coculture in the presence of 2.5 mM of glufosinate and 12.5 mM of glutamine.

To further verify that the inhibitory effect of glufosinate is due to the inhibition of the GlnS enzyme, we tried to bypass the inhibition via glutamine supplementation, the product of GlnS. The inhibitory effect of glufosinate on *P. sedebokerense* infection development in *H. lacustris* cultures was abolished by the addition of 12.5 mM glutamine. An infection of *H. lacustris* develops in the presence of 2.5 mM of glufosinate with the amino acid added but remains inhibited at this concentration if the amino acid is not added (Figure 3). We also tried to measure GlnS activity in preparations of both pure propagules and a logarithmic culture of *P. sedebokerense* but failed to detect any activity. We tried both the transferase GlnS assay [32] and the biosynthetic GlnS assay [33]. In plants, bacteria, and animals it is widely accepted that the GlnS is regulated at the level of transcription, posttranscription, translation, and post translation. Though little is known about the regulatory mechanism of GlnS activity in fungi [43], one of those mechanisms might be the reason why we could not detect GlnS activity in *P. sedebokerense*. We thus searched for the GlnS gene in the draft genome (unpublished) of our *P. sedebokerense* strain.

### 3.3. Glutamine Synthetase of P. sedebokerense

**Figure 4:**
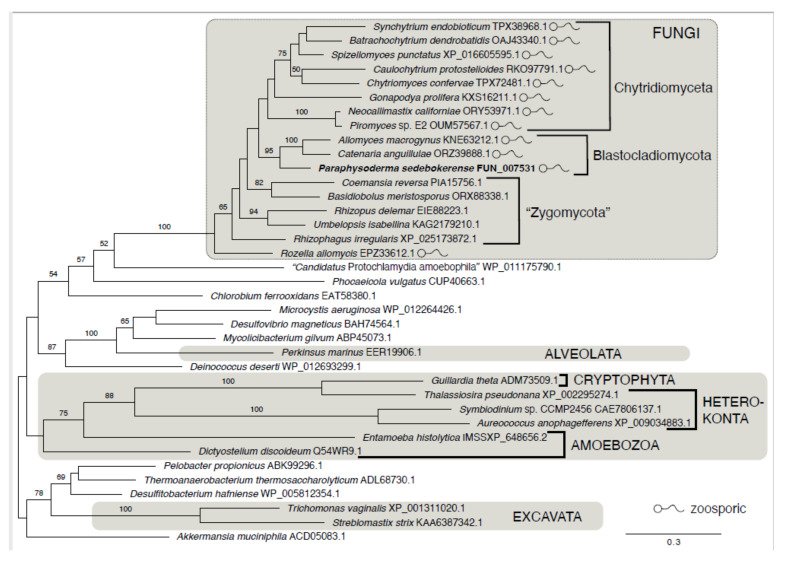
Phylogeny of glutamine synthetase type III proteins shows monophyly of fungal copies and a distribution restricted to early diverging lineages of fungi. Clades of eukaryotes are indicated by a grey background, and other sequences are from bacteria. Only bootstrap values of 50% are shown.

We found a putative protein of 756 amino acids (GenBank OM687488) within the predicted proteome of *P. sedebokerense*, which showed a similarity of 74% with GlnS type III of *Catenaria anguillullae*. We constructed a phylogenetic tree with GlnS type III of other fungi and the GlnS type III of *P. sedebokerense* sequence, which formed a clade strongly supported (BS 95) with *C. anguillullae* and *Allomyces macrogynus* and related to another fungal GlnS type III, suggesting that our putative protein sequence is also a GlnS type III (Figure 4).

### 3.4. Effect of Glufosinate on the Growth of P. sedebokerense

**Figure 5:**
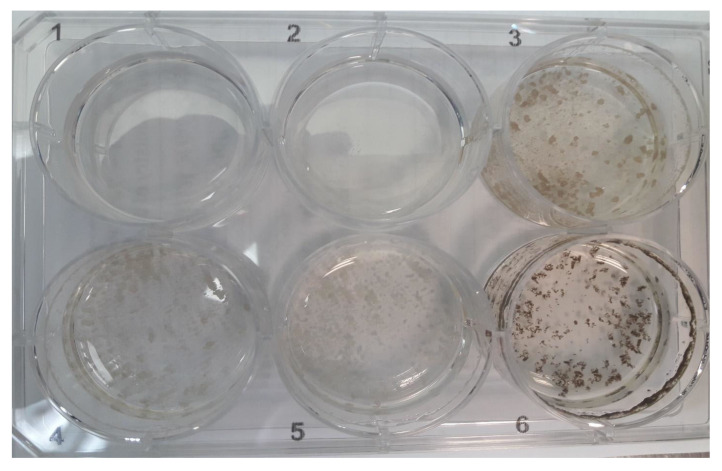
Inhibition *P. sedebokerense* growth in synthetic medium by increasing concentrations of glufosinate. Purified propagules (7 × 10^−5^ cells mL^−1^) were inoculated in BGM medium with 2.5 (1), 1.25 (2), 0.5 (4), and 0.1 (5) mM glufosinate or without glufosinate (3, negative control); 6, coculture of *P. sedebokerense* and *Haematococcus* without glufosinate to show blastoclad infectivity. All cultures were incubated at 30 °C for 3 days.

The effect of glufosinate on the blastoclad growth is evident when *P. sedebokerense* propagules were inoculated with glufosinate in a rich culture medium (BGM) such as saprobe. High concentrations of glufosinate—1.25 and 2.5 mM—completely inhibited the growth, whereas lower concentrations of 0.1 and 0.5 mM reduced the growth of blastoclads, as compared with the non-treated control culture (Figure 5).

### 3.5. Growth of P. sedebokerense on Different Nitrogen Sources

**Figure 6:**
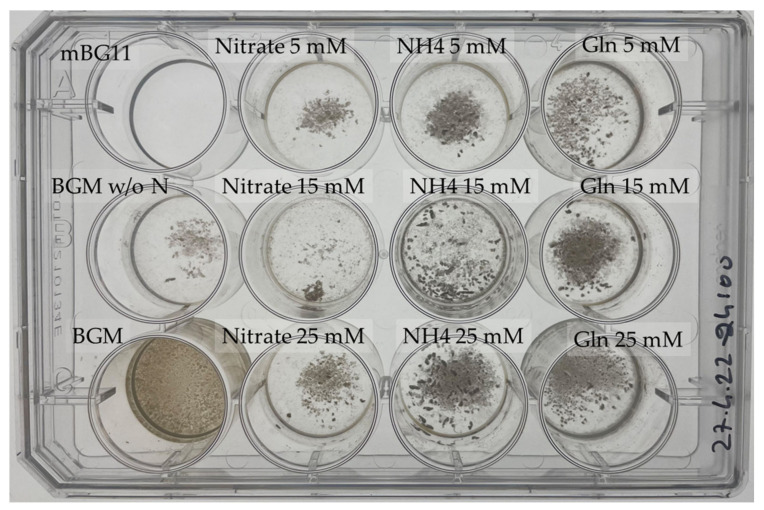
*P. sedebokerense* growth in culture media supplemented with nitrate, ammonia, and glutamine at 5, 15, and 25 milli equivalents of N. propagules (2 × 10^5^ cells), respectively, and inoculated in 2 mL of BGM w/o nitrogen. The left column: BGM is the positive control and BGM w/o N and mBG are the negative controls; nitrate, ammonia, and glutamine (left to right, from second to fourth column) were added at 5, 15, and 25 milli equivalents of N (top to bottom), respectively, and cultures were grown for 10 days.

We tested the ability of *P. sedebokerense* to grow on nitrate, ammonia, and glutamine as nitrogen sources, at three different concentrations. Our results (Figure 6) show that *P. sedebokerense* assimilates nitrogen from inorganic (NaNO_3_ and NH_4_) and organic (Gln and BGM) nitrogen sources. Starting from isolated propagules, we observed the development of blastoclad biomasses in the three assayed nitrogen sources, with a trend of higher biomass development in the higher concentration (25 > 15 > 5 mM). We used two different negative controls, mBG11, which doesn’t contains nitrogen source at all, and BGM w/o N, which contains nitrogen at trace level (0.5 mM) and sucrose. We found poor growth in BGM w/o N and in low nitrate concentrations (5 and 15 mM), but absolutely no growth in mBG11. We also found low growth in ammonia and glutamine at a low concentration (5 mM). The growth with nitrate at a higher concentration (25 mM) is between poor and low, but statistically different from BGM w/o N since the confidence intervals at 95% are not overlapping (Table 1). In the cultures supplemented with high concentrations (15 and 25 mM) of glutamine and ammonia, we found high growth, which is statistically higher than the treatments, but lower than the positive control (Figure 6). These results show that the blastoclad prefers complex nitrogen sources, such as those present in peptone (BGM), rather than simple nitrogen sources, as with the treatments. High nitrogen concentrations are mandatory for intensive growth—15 and 25 mM supported high growth, whereas 5 mM showed low growth. The three different compounds assayed support growth; however, nitrate showed much lower growth, and only 25 mM showed statistically significant growth as compared to the negative control (Table 1).

## 4. Discussion

We studied the effect of GlnS inhibitor on *P. sedebokerense* infection development in *H. lacustris* cultures and found a complete inhibition of the infection, both in the green and red stages of culture, with 2.5 mM glufosinate. In the presence of glufosinate, inoculated propagules of *P. sedebokerense* encysted on *H. lacustris* cells but did not develop or propagate further, and thus progression of the infection to culture collapse was prevented (Figure 2). This inhibitory effect is not due to the inability to mobilize nitrogen compounds from the host, since the *H. lacustris* cells which were pretreated (irreversibly) with glufosinate were susceptible to *P. sedebokerense* infection to the same extent as the negative control non-treated cells (Figure 1). We showed that glufosinate inhibited the growth of *P. sedebokerense* as a saprotroph (Figure 4), and we provided evidence that this effect is due to the blockage of GlnS, since the addition of glutamine reverts the inhibitory effect of glufosinate on the infection of *H. lacustris* (Figure 3). We failed to detect fungal GlnS enzyme activity, although we used different assays. Little is known about the regulatory mechanism of GlnS activity in fungi; however, it was recently shown that transcriptional and posttranslational modifications are involved in the regulation of GlnS activity in the fungus *Ganoderma lucidum* [43]. We assume that in *P. sedebokerense,* GlnS is active during a specific growth stage which we could not identify yet. Biosynthesis inhibitors of other amino acids have proven to be effective in controlling *P. sedebokerense* infection, i.e., methionine biosynthesis inhibitors [17]. GlnS plays a central role in the nitrogen metabolism of fungi [43,44], because it converts inorganic nitrogen (in the form of ammonia) to organic nitrogen in the form of glutamine [27,28,45], from which all amino acids are produced; consequently, GlnS should be a major target to control the disease to *H. lacustris*. We found that the GlnS present in the genome of *P. sedebokerense* is a GlnS type III. This GlnS type is the least characterized of the three described isozymes. Here we show that GlnS type III is present in many of the poorer known phyla of fungi, including Blastocladiomycota and Chytridiomycota, and absent in the main subkingdom of fungi, the Dikarya. All types of GlnS have conserved catalytic domain, but type III is different and bigger than GlnS type I and GlnS type II, which are present in *H. lacustris* and other microalgae [46]. Glufosinate is an effective inhibitor of GlnS and irreversibly blocks the three types of GlnS; however, glufosinate affects the fungal parasitoid and its microalgal prey differently, i.e., it kills the fungus but only inhibits the host growth. Additional knowledge and studies on GlnS sequence in *P. sedebokerense* and other algal parasites can help to develop strategies to control algal diseases. Future studies could discover or design GlnS type III-specific inhibitors that affect only GlnS of *P. sedebokerense* but not GlnS of *H. lacustris*.

Our results show that *P. sedebokerense* can utilize organic and inorganic nitrogen sources, and the best growth promoting nitrogen sources (supplemented to mBG_11_ + sucrose + YE w/o N) are ammonia and glutamine at 25 and 15 mM (Figure 6, Table 1).

The ability of *P. sedebokerense* to use different nitrogen sources, including inorganic nitrogen sources, is in agreement with recent findings [17] but in contradiction with the results of Hoffman et al. [1]. The reason why Hoffman et al. failed to detect growth with the inorganic nitrogen sources nitrate and ammonia may be related to the lack of vitamins that were supplied in our assays in the form of yeast extract without amino acids but were absent in the previous trials. Previous results showing that nitrate facilitates *P. sedebokerense* infection development in *H. lacustris* and propagule production is induced in its absence (19) are in accordance with the blastoclad ability to use nitrate, as shown in this study.

Our findings of the use of nitrate are in accordance with the results of Digby et al. [26], who showed that most of the zoosporic true fungi could use nitrate as their sole nitrogen source. Even more, Lin et al. [17] showed how during the encystmet and penetration of *H. lacustris* by *P. sedebokerense*, nitrate assimilation genes (including nitrate reductase and nitrate transmembrane transporter) are overexpressed. On the other hand, Digby et al. [26] specifically failed to grow on nitrate the two Blastocladiomycota species. Similarly, Nolan [47] and Theodorou et al. [48] also reported the inability of Blastocladiomycota to grow on nitrate. We assume that this could be due to the absence of another limiting factor, as also happened with *P. sedebokerense* in Hoffman et al. [1]. The observed higher efficiency in the use of ammonia as compared with nitrate is highly expected, since ammonia is the best nitrogen source for some fungi [28,49], and it requires fewer steps (and less energy) to be assimilated (see the KEGG reference pathway of nitrogen metabolism for fungi [45]. Our results are in agreement with previous works since ammonia is the best nitrogen source for some fungi [28,49], and it requires two fewer steps (and less energy) than nitrate to be assimilated (see the KEGG reference pathway of nitrogen metabolism for fungi [45]). However, these results are different from those of Lin et al. (17), who reported equal capability to utilize ammonia and nitrate, based on their experiment with Phenotype MicroArray plates; this experimental system can probably be used as a proxy, but must be carefully verified, as demonstrated in the current study.

Our results, together with those of Lin et al. [17], show that although *P. sedebokerense* is able to grow when nitrogen is supplied as nitrate, ammonia, or glutamine, the success is lower than growing the fungi in a BGM/ECGM medium (containing peptone and yeast extract). Our results provided evidence for the importance of the GlnS enzyme in the nitrogen metabolism of *P. sedebokerense* since the addition of glufosinate to cultures inhibits the growth of the blastoclad despite the presence of various amino acids and peptides in the full BGM medium. The literature on nitrogen metabolism of eukaryotes in general and fungi in particular points to GlnS as the pivotal enzyme of nitrogen metabolism, since it is the key step linking the assimilation of inorganic nitrogen with amino acid synthesis [27,28]. The effect of glufosinate on *P. sedebokerense* is similar on both saprobic and parasitoid lifestyles, but the threshold for complete inhibition is doubled in the parasitoid lifestyle (2.5 mM) as compared with the saprobic lifestyle (1.25 mM). We assume that this threshold difference could be due to the fact that glufosinate is partly taken up by the host, and as an irreversible inhibitor it stays occluded inside the bigger host cells, causing a significant decrease in the extracellular concentration of glufosinate. The effect of glufosinate on *H. lacustris* was demonstrated in detail previously [29]. We have confirmed that the effect of glufosinate on *P. sedebokerense* is because it blocks the GlnS and prevents the synthesis of glutamine, since the effect reverts when glutamine is added.

In summary, our data point to the importance of GlnS in controlling the growth and virulence of *P. sedebokerense*, and we determined the GlnS of *P. sedebokerense* to belong to type III, which is only found in non-Dikarya fungi. Using nitrogen-free culture well plates and the supply of vitamins to avoid limiting factor deficiency, we confirmed the ability of *P. sedebokerense* to use inorganic nitrogen; moreover, we showed that the three main nitrogen compounds of the nitrogen assimilation pathway promote the growth of the blastoclad, being ammonia and glutamine, and the substrate and the product of Glutamine synthetase to be the best growth-promoting compounds. The fact that glufosinate increases the intracellular concentration of ammonia [29] and ammonia promotes the growth of *P. sedebokerense* reinforces the conclusion that the inhibition of blastoclad growth caused by glufosinate is due to inhibition of Glns and suggests a central role for this enzyme in the blastoclads’ nitrogen metabolism.

We can conclude that the blastoclad *P. sedebokerense* can utilize more nitrogen sources than previously reported [1]. This capability of growth using two of the most common nitrogen sources in environmental waters, such as ammonia and nitrate, is on one hand related to the ability to survive as a saprobe, and more environments could be a reservoir. On the other hand, our results shows that Glns synthetase is an efficient target to control *P. sedebokerense* as microalgal pest. Currently, with the herbicide glufosinate, the infection to *H. lacustris* can be controlled at the expense of inhibiting the microalgal growth but facilitating the production of astaxanthin. In addition, we expect that further strategies based on specific inhibitors to Glns type III will be developed to inhibit the fungal pest but not the microalgal hosts.

## Figures and Tables

**Table 1:** Wet weight of *P. sedebokerense* cultures inoculated with different nitrogen sources obtained after 10 days of growth (values are expressed in mg). Data were obtained from the experiment shown in Figure 6. Asterisk symbols refer to statistical differences as non-overlapping confidence intervals.

	Replicate 1	Replicate 2	Replicate 3	Average	STDEV	CI 95 Lower	CI 95 High	Statistical Differences
mBG11 N-	0.6	0.2	0.6	0.47	0.20	0.12	0.82	Negative control
Full BGM	84.3	80	74.8	79.70	2.57	75.30	84.10	Positive control
BGM w/o N	9.9	12.2	10.9	11.00	1.15	9.03	12.97	*
5 mM Nitrate	8.9	13.8	10.4	11.03	2.46	6.83	15.24	*
15 mM Nitrate	14	18	13.4	15.13	2.06	11.61	18.66	*
25 mM Nitrate	15.8	22.6	18.9	19.10	3.40	13.28	24.92	*/**
5 mM Ammonia	22.9	21.9	30.2	25.00	1.58	22.29	27.71	**
15 mM Ammonia	38.6	39.1	44.6	40.77	1.13	38.83	42.71	***
25 mM Ammonia	42.1	42.4	41.7	42.07	0.18	41.75	42.38	***
5 mM Gln	23.4	29.4	20.9	24.57	3.18	19.12	30.01	**
15 mM Gln	55.6	39.6	48.8	48.00	8.00	34.31	61.69	***
25 mM Gln	37.2	42.6	53.8	44.53	3.80	38.03	51.04	***

## Data Availability

Data is contained within the article and the sequence *of P. sedebokerense* Glns is submitted in GenBank with accession number OM687488.

## Supplementary Materials

The following supporting information can be downloaded at: https://www.mdpi.com/article/10.3390/jof8060561/s1, Figure S1: Inhibition of *H. pluvialis* infection by *P. sedebokerense* at different dose of glufosinate.
