# Supplementary material for: Paraphysoderma sedebokerense GlnS III Is Essential for the Infection of Its Host Haematococcus lacustris
Source: J Fungi (Basel). 2022 May 25;8(6):561. doi: 10.3390/jof8060561 (PMC9224648; doi:10.3390/jof8060561)
Supplement: Supplementary file 1 [file jof-08-00561-s001.zip › Supplementary.pdf]

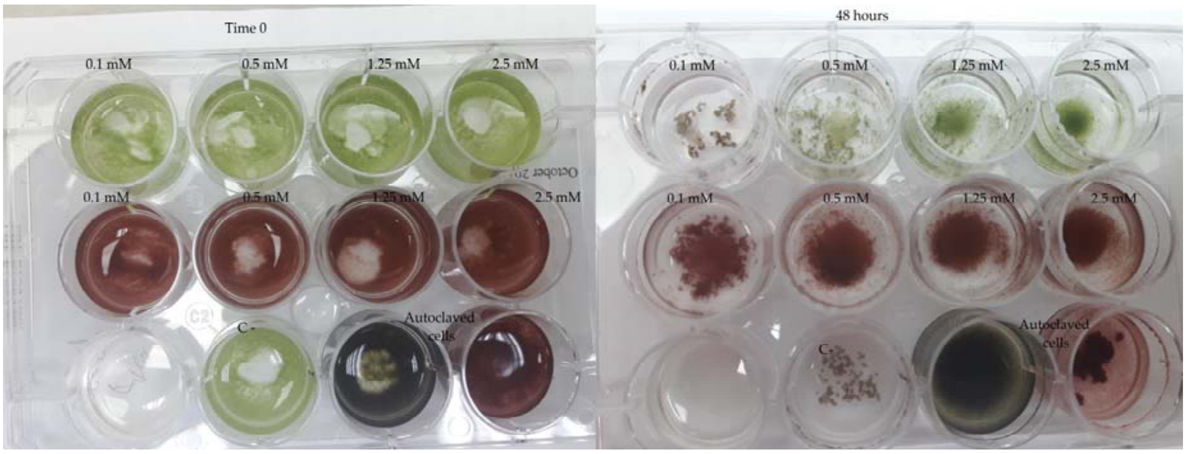

Figure S1: Inhibition of *H. pluvialis* infection by *P. sedebokerense* at different dose of glufosinate.
